# Xeno-free culture of human pluripotent stem cells on oligopeptide-grafted hydrogels with various molecular designs

**DOI:** 10.1038/srep45146

**Published:** 2017-03-23

**Authors:** Yen-Ming Chen, Li-Hua Chen, Meng-Pei Li, Hsing-Fen Li, Akon Higuchi, S. Suresh Kumar, Qing-Dong Ling, Abdullah A. Alarfaj, Murugan A. Munusamy, Yung Chang, Giovanni Benelli, Kadarkarai Murugan, Akihiro Umezawa

**Affiliations:** 1 Department of Chemical and Materials Engineering, National Central University, No. 300, Jhongda RD., Jhongli, Taoyuan, 32001 Taiwan; 2 Department of Reproduction, National Research Institute for Child Health and Development, 2-10-1 Okura, Setagaya-ku, Tokyo 157-8535, Japan; 3 Department of Botany and Microbiology, College of Science, King Saud University, Riyadh 11451, Saudi Arabia; 4 Department of Medical Microbiology and Parasitology, Universiti Putra Malaysia, 43400 Serdang, Slangor, Malaysia; 5 Cathay Medical Research Institute, Cathay General Hospital, No. 32, Ln 160, Jian-Cheng Road, Hsi-Chi City, Taipei, 221, Taiwan; 6 Graduate Institute of Systems Biology and Bioinformatics, National Central University, No. 300, Jhongda RD., Jhongli, Taoyuan, 32001 Taiwan; 7 Department of Chemical Engineering, R&D Center for Membrane Technology, Chung Yuan Christian University, 200, Chung-Bei Rd., Chungli, Taoyuan, 320, Taiwan; 8 Department of Agriculture, Food and Environment, University of Pisa, Via del Borghetto 80, 56124 Pisa, Italy; 9 Division of Entomology, Department of Zoology, School of Life Sciences, Bharathiar University, Coimbatore, Tamil Nadu, 641 046, India; 10 Department of Zoology, Thiruvalluvar University, Serkkadu, Vellore 632 115, India

## Abstract

Establishing cultures of human embryonic (ES) and induced pluripotent (iPS) stem cells in xeno-free conditions is essential for producing clinical-grade cells. Development of cell culture biomaterials for human ES and iPS cells is critical for this purpose. We designed several structures of oligopeptide-grafted poly (vinyl alcohol-co-itaconic acid) hydrogels with optimal elasticity, and prepared them in formations of single chain, single chain with joint segment, dual chain with joint segment, and branched-type chain. Oligopeptide sequences were selected from integrin- and glycosaminoglycan-binding domains of the extracellular matrix. The hydrogels grafted with vitronectin-derived oligopeptides having a joint segment or a dual chain, which has a storage modulus of 25 kPa, supported the long-term culture of human ES and iPS cells for over 10 passages. The dual chain and/or joint segment with cell adhesion molecules on the hydrogels facilitated the proliferation and pluripotency of human ES and iPS cells.

People sustain damage or loss of tissues and organs from diseases, birth defects, or accidents. Human embryonic stem (ES) cells and human induced pluripotent stem (iPS) cells are attractive cell sources for drug discovery and regenerative medicine^1^,^2^,^3^,^4^,^5^. Human pluripotent stem cells (human ES and iPS cells) have the potential to differentiate into any kind of cell and produce an unlimited cell source for cell therapy^6^,^7^,^8^,^9^,^10^. However, human ES and iPS cells require special cell culture environments to maintain their pluripotency, and these stem cells cannot be cultured on conventional tissue culture polystyrene (TCPS) dishes due to their high differentiation ability^2^,^11^,^12^,^13^,^14^. Therefore, developing biomaterials for culturing human ES and iPS cells while maintaining their pluripotency is an important topic of research. Typically, human ES and iPS cells are cultivated on mouse embryonic fibroblasts (MEFs) under xeno-containing and feeder cell conditions, or on Matrigel-coated dishes as a feeder-free but not xeno-free condition^2^. Xeno-free growth conditions are required for clinical application of cultured cells. Recently, recombinant vitronectin (rVitronectin)-coated dishes were used for human ES and iPS cell culture as a xeno-free culture medium^15^. Recombinant vitronectin is an extracellular matrix (ECM) protein, typically produced by fermenting genetically recombinant *E. coli*. However, recombinant vitronectin is relatively expensive to mass produce. Inexpensive and completely synthetic cell culture dishes are necessary to safely produce human ES and iPS cells for clinical application, because large numbers of stem cells are needed for patient treatment (typically 10^6^–10^7^ cells/kg)^16^,^17^,^18^.

Several completely synthetic cell culture materials for human ES and iPS cells have been designed, such as dishes grafted with ECM-derived oligopeptides^11^,^19^,^20^,^21^,^22^,^23^,^24^,^25^ and dishes made of synthetic polymers^13^,^26^,^27^,^28^,^29^. Completely synthetic dishes, such as (a) copoly[2-(acryloyloxyethyl)] trimethylammonium-co-2-(diethylamino)ethyl acrylate, copoly(AEtMA-co-DEAEA)^26^, (b) aminopropylmethacrylamide, APMAAm^27^, (c) poly[2-(methacryloyloxy](ethyldimethyl-(3-sulfopropyl)ammoniumhydroxide, PMEDSAH^13^,^28^, and (d) poly(methyl vinyl ether-alt-maleic anhydride), PMVE-alt-MA^29^, have been used to culture human ES and iPS cells in chemically defined media. However, it is necessary to evaluate these heparin-mimicking biomaterials to determine the reproducibility of data reported by other research groups, because the cellular binding of human ES and iPS cells to these polymeric materials is expected to be too weak to maintain the pluripotency of these human ES and iPS cells during culture. Currently, the most reliable materials for culturing human ES and iPS cells, which are synthetic and not produced by fermentation, are materials grafted with ECM-derived oligopeptides^2^.

Polyacrylate surfaces grafted with bioactive oligopeptides using 1-ethyl-3-(3-dimethylaminopropyl)-carbodiimide/N-hydroxysuccinimide (EDC/NHS) chemistry was first reported by Melkoumian *et al*.^25^. Human ES cells (H1 and H7 lines) were successfully cultured and expanded on a polyacrylate surface grafted with bone sialoprotein oligopeptide (BSP) and VN (vitronectin)-derived oligopeptides, which have a fixed elasticity, in xeno-free medium. The oligopeptide surface density is also important, with oligopeptide concentrations of 0.25–1 mM (393–1,572 μg/mL) being necessary to maintain human ES cell colonies on oligopeptide surfaces^25^.

The cyclic RGD (arginine-glycine-aspartic acid) peptide, CRGDC expresses significantly higher cell-binding ability than the conventional linear GRGDSP peptide via the α_5_β_1_, α_V_β_5_, and α_V_β_3_ integrins, where the α_5_β_1_ and α_V_β_5_ integrins are typically used for attaching human ES and iPS cells on ECMs^30^,^31^. Therefore, cyclic RGD peptide-grafted surfaces were designed for human ES cell culture by Kolhar *et al*.^24^. Cyclic RGD peptides were immobilized on an amine-modified surface with a bifunctional linker that binds with a thiol of the peptide and an amine group on the surface at 10–30 fmol of cyclic RGD peptide/cm^2^. Human ES cells (H14 and H9 lines) were cultured on cyclic RGD peptide-conjugated surfaces and pluripotency was maintained in MEF-conditioned (xeno-containing) medium for 10 passages^24^. This study did not optimize the elasticity of cell culture materials that are grafted with cyclic RGD peptides.

Self-assembled monolayers of oligopeptide-conjugating alkanethiols, which maintain the pluripotency of human ES cells, were developed by Klim *et al*.^21^. Eighteen types of specific oligopeptide-conjugated alkanethiols were designed. Monolayers composed of heparin-binding oligopeptides (GWQPPARARI (fibronectin domain), FHRRIKA (bone sialoprotein domain), and GKKQRFRHRNRKG (vitronectin domain)) promoted adhesion and proliferation of human ES cells (H9) in chemically defined (mTeSR1) medium with the addition of Y-27632 (ROCK inhibitor), whereas monolayers containing the integrin ligand KGRGDS did not maintain human ES cell pluripotency^21^. Specific oligopeptide-immobilized monolayers supported human ES cell culture and pluripotency under a chemically defined medium in this study, although they did not optimize the elasticity of cell culture materials.

Park *et al*. developed bioactive oligopeptide-conjugated polydopamine as a coating material on several different polymeric surfaces based on bio-inspiration from the strong adhesion ability of mussels. This adhesion results from repeating units of amine (lysine) and L-Dopa (3,4-dihydroxy-L-phenylalanine) residues in the *Mytilus edulis* foot protein-5 of mussel adhesive pads^11^. Human ES and iPS cells were successfully cultivated on surfaces coated with oligopeptide-conjugated polydopamine, where the oligopeptide moiety was derived from vitronectin conjugated with or without cysteine residues to generate single or dual chains, respectively. Surfaces coated with oligopeptide-conjugated polydopamine had significantly decreased elastic moduli^11^. Human iPS cells cultivated on surfaces coated with dual-chain oligopeptide-conjugated polydopamine expressed the focal adhesion protein vinculin and had organized cytoskeletal elements (F-actin), leading to greater colony attachment of human iPS cells compared to colony attachment on single chains of oligopeptides. Human ES and iPS cells can be cultivated on surfaces coated with oligopeptide-conjugated polydopamine for 15 passages in feeder-free conditions^11^. However, long-term culture of human ES or iPS cells was not observed on surfaces coated with oligopeptide-conjugated polydopamine in xeno-free conditions. Furthermore, the effect of cell culture material elasticity on human ES and iPS cell culture was not investigated in this study.

In our previous study^12^, we designed biomaterials for culturing human ES and iPS cells based on the combination of physical cues (biomaterial elasticity) and biological cues (specific cell adhesion molecules). Polyvinyl alcohol-co-itaconic acid (PVA) hydrogels were grafted with an oligopeptide derived from vitronectin (KGGPQVTRGDVFTMP) to promote the adhesion of human ES and iPS cells to the hydrogels, and the elasticity (storage modulus, *E’*) of the hydrogels was adjusted from 10–30 kPa by altering the crosslinking time. It was difficult for human ES cells to attach to the soft hydrogels (10 kPa)^12^. Human ES cells grown on the stiffest hydrogels (30.4 kPa) differentiated after few days of cultivation, whereas human ES and iPS cells cultivated on hydrogels with the optimal elasticity (25 kPa) supported proliferation and pluripotency for more than twenty passages in xeno-free cell culture conditions. The previous data indicate that the stiffness of cell culture biomaterials is important for maintaining the pluripotency of human ES and iPS cells^12^. However, in this previous study, human ES cells and iPS cells were cultivated on hydrogels with different elasticity but with only one specific oligopeptide (VN1). Currently, it is not clear which molecular design of oligopeptides is preferable for grafting onto hydrogels with optimal elasticity.

Therefore, we designed oligopeptide-grafted hydrogels with several structures: single chains, single chains with joint segment, dual chains with joint segment, and branched-type chains. Furthermore, oligopeptide sequences used for oligopeptide-grafted hydrogels were selected from the integrin-binding domain of ECM and the glycosaminoglycan-binding domain. The goal of this study was to investigate which oligopeptide-grafted hydrogel molecular design and elasticity favorably supports long-term culture of human ES and iPS cells while maintaining their pluripotency in xeno-free cell culture conditions.

## Results

### Preparation and characterization of PVA hydrogels grafted with various oligopeptides

**Figure 1:**
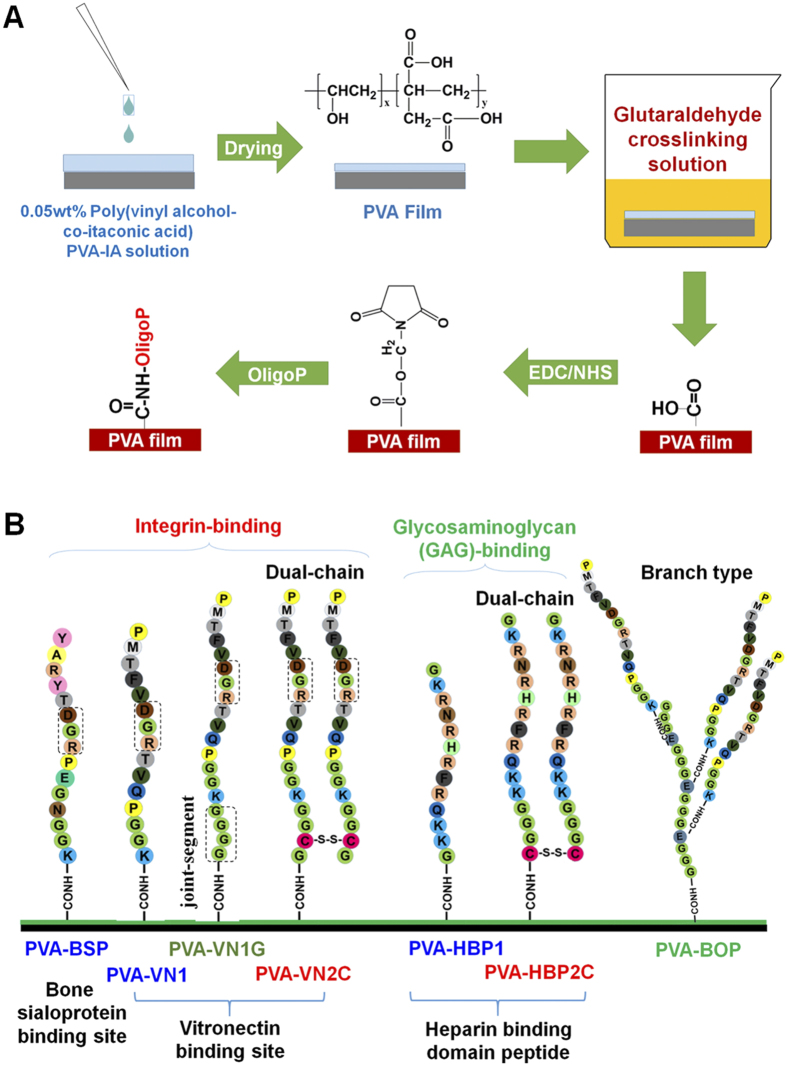
Preparation and design of PVA hydrogels grafted with several oligopeptides containing cell-binding domains. (**A**) A chemical scheme for PVA hydrogels reacting with oligopeptides (oligoP). (**B**) Design and sequence of oligopeptides grafted on PVA hydrogels. Single chains (PVA-BSP, PVA-VN1, and PVA-HBP1), single chains with a joint segment (PVA-VN1G), dual chains (PVA-VN2C and PVA-HBP2C), and branch-type (PVA-BOP) oligopeptides were grafted on PVA hydrogels. PVA-BSP, PVA-VN1, PVA-VN1G, PVA-VN2C, and PVA-BOP have an integrin-binding domain, whereas PVA-HBP1 and PVA-HBP2C have a glycosaminoglycan-binding domain.

Poly(vinyl alcohol-co-vinyl acetate-co-itaconic acid) was used to prepare PVA hydrogels grafted with various oligopeptides in this study. This is because poly(vinyl alcohol-co-vinyl acetate-co-itaconic acid) has carboxylic moiety to bind the oligopeptides and can be commercially available. PVA hydrogels were grafted with several oligopeptides in various patterns, structures of which are shown in Fig. 1B. It should be noted that BSP, VN1, VN1G, VN2C, HBP1, and HBP2C contain basic side chain of amino acids (lysine and arginine). Therefore, there is another reaction possibility to graft the oligopeptides on PVA hydrogels via side chain of lysine or arginine in the oligopeptides and not only to graft the oligopeptides on PVA hydrogels via end amino group of the oligopeptides shown in Fig. 1B. The elasticity of PVA hydrogels was regulated by altering the time of crosslinking in the glutaraldehyde crosslinking solution (Fig. 1A). PVA films with a thickness of 1.3–1.6 μm under dry conditions were selected for these experiments. In our previous study^12^, PVA hydrogels grafted with VN1, which showed an *E*’ of 25 kPa, were found to be the most optimal hydrogels, as they supported the proliferation of human ES and iPS cells. Therefore, PVA hydrogels prepared by crosslinking for 24 h and showing an *E*’ of 25 kPa were used for the base hydrogels to be grafted with different oligopeptide patterns (Fig. 1B).

**Figure 2:**
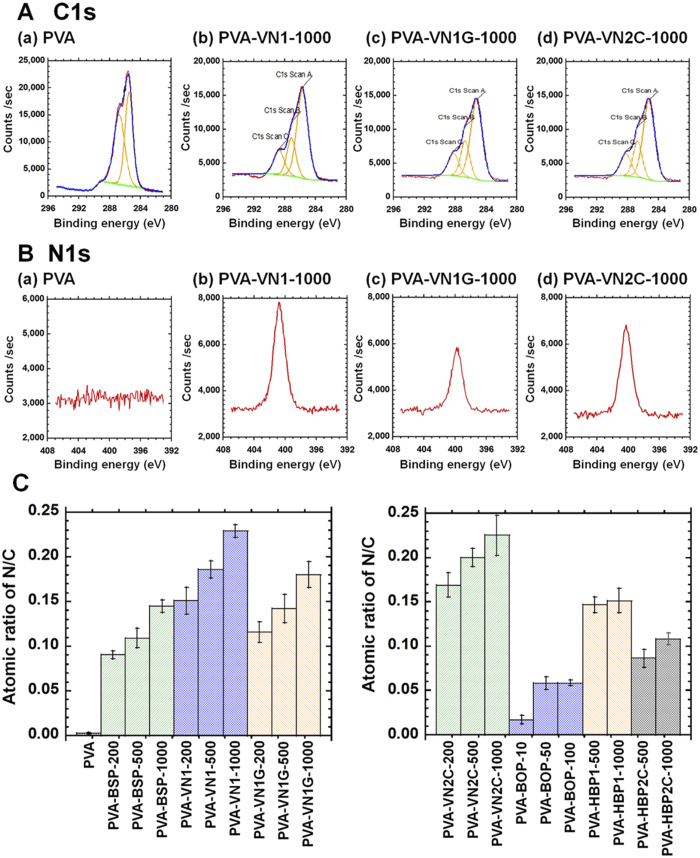
Characterization of PVA hydrogels grafted with various oligopeptides. (**A**) High-resolution XPS spectra of the C1s peaks analyzed on the surface of unmodified PVA (a), PVA-VN1-1000 (b), PVA-VN1G-1000 (c), and PVA-VN2C-1000 (d) hydrogels. (**B**) High-resolution XPS spectra of the N1s peaks analyzed on the surface of unmodified PVA (a), PVA-VN1-1000 (b), PVA-VN1G-1000 (c), and PVA-VN2C-1000 (d) hydrogels. (**C**) The nitrogen to carbon (N/C) atomic ratios in PVA and PVA-BSP, PVA-VN1, PVA-VN1G, PVA-VN2C, PVA-BOP, PVA-HBP1, and PVA-HBP2C hydrogels grafted with different concentrations of oligopeptides (200, 500, or 1000 μg/mL).

It is important to analyze the effects and surface densities of oligopeptides on oligopeptide-grafted PVA hydrogels with different oligopeptide structures. However, it was difficult to quantify the amount of oligopeptides grafted on PVA hydrogels via chemical titration or reaction methods in this study. Furthermore, the absolute quantity of oligopeptides on the oligopeptide-grafted PVA hydrogels cannot be analyzed using an enzyme-linked immunosorbent assay (ELISA). Therefore, we investigated the surface of oligopeptide-grafted PVA hydrogels using X-ray photoelectron spectroscopy (XPS). Figure 2A,B show the high-resolution XPS spectra of the C1s and N1s peaks, respectively, of unmodified PVA hydrogels (PVA) and oligopeptide-grafted PVA hydrogels (PVA-VN1, PVA-VN1G, and PVA-VN2C) prepared with 1000 μg/mL oligopeptide solutions where PVA-*X*-*Y* indicates hydrogels grafted with oligopeptide *X* (BSP, VN1, VN1G, VN2C, HBP1, or HBP2C) using *Y* (200, 500, or 1000) μg/mL of the oligopeptide solution. The XPS spectra of the C1s and N1s peaks on oligopeptide-grafted PVA hydrogels (PVA-BSP, PVA-VN1, PVA-VN1G, PVA-VN2C, and PVA-BOP), prepared with 200 or 500 μg/mL oligopeptide solutions, are shown in Supplementary Figs 1 and 2.

C–H and C–C bonding (285 eV), O–C = O bonding (289 eV), and C–N bonding (286 eV) were clearly observed in the XPS spectra of oligopeptide-grafted PVA hydrogels compared to the unmodified PVA hydrogels (Fig. 2A(b–d)). In contrast, C–H and C–C bonding (285 eV) were mainly observed in the XPS spectra of the unmodified PVA hydrogels (Fig. 2A(a)). These results indicate that oligopeptides were covalently immobilized on the PVA hydrogels.

The high-resolution XPS spectra of the N1s peaks were evaluated for oligopeptide-grafted PVA hydrogels and unmodified PVA hydrogels. An N1s peak around 400 eV was clearly detected on the oligopeptide-grafted PVA hydrogels (Fig. 2B(b–d)), which originated from the nitrogen atoms of amino acids in oligopeptides, whereas no N1s peak was observed on unmodified PVA hydrogels (Fig. 2B(a)), owing to the fact that initially PVA hydrogels do not have any nitrogen atoms.

The N/C atomic ratios of oligopeptide-grafted PVA hydrogels and unmodified PVA hydrogels were determined from the XPS spectra, and the results are shown in Fig. 2C. The N/C ratios of unmodified PVA showed minimal values (negative control). The branch-type oligopeptide structures (BOP-10, BOP-50, and BOP-100) showed relatively low N/C ratios compared to other oligopeptide-grafted PVA hydrogels where PVA-BOP-*Z* indicates hydrogels grafted with the main chain using *Z* (10, 50, or 100) μg/mL solution. These results were possibly obtained by the dual activation process using NHS/EDC, which might damage the grafted PVA hydrogel surfaces. N/C ratios increased for oligopeptide-grafted PVA hydrogels prepared from any single oligopeptide chain (PVA-BSP, PVA-VN1, PVA-VN1G, and PVA-HBP1) or dual oligopeptide chain (PVA-VN2C and PVA-HBP2C) with increasing oligopeptide concentrations. However, the observed increase in N/C ratio of PVA hydrogels grafted with any oligopeptide using 1000 μg/mL oligopeptide solution showed a less than 0–15% increase in N/C ratio compared to PVA hydrogels grafted with the corresponding oligopeptide at 500 μg/mL. From these results, grafting of single or dual oligopeptide chains on PVA hydrogels using NHS/EDC chemistry seemed to approach saturation when using oligopeptide solutions of 500–1000 μg/mL.

### Culture of human ES cells on oligopeptide-grafted PVA hydrogels prepared from different oligopeptide designs

**Figure 3:**
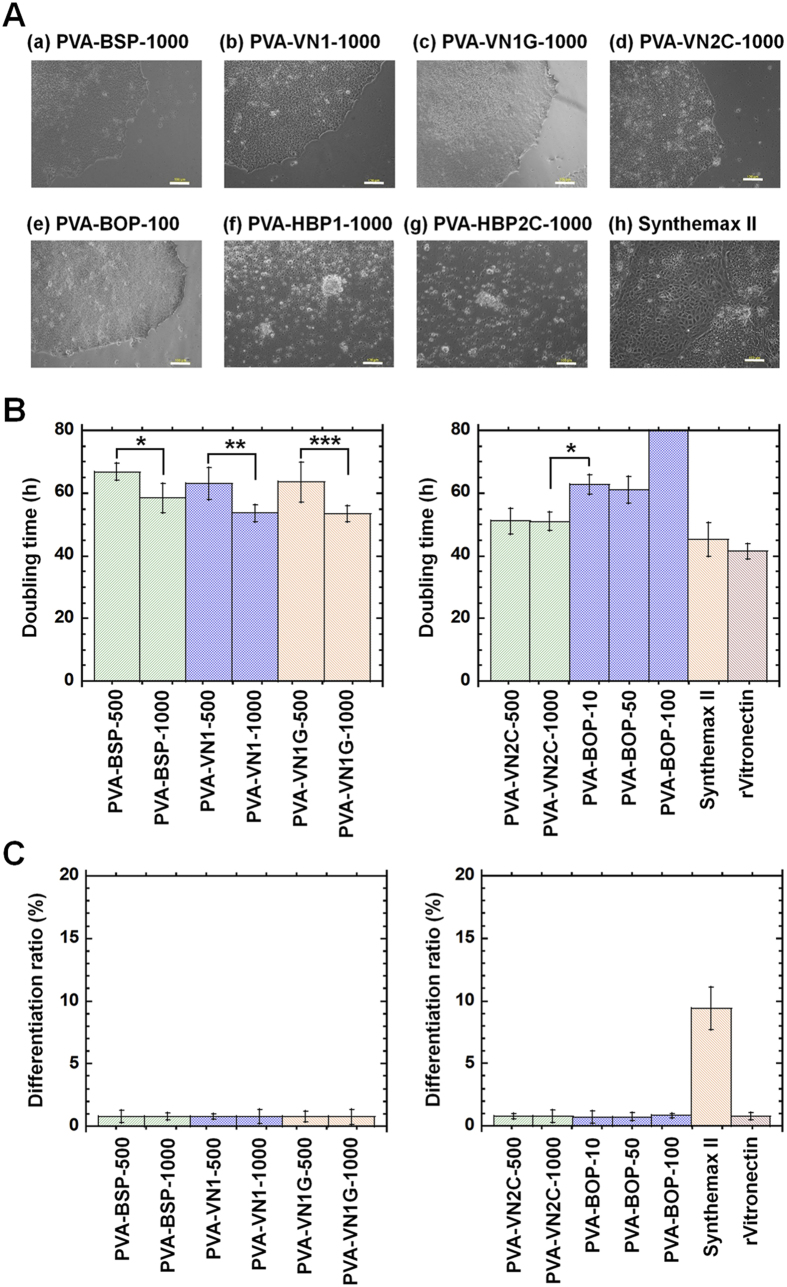
Human ES cells cultured on PVA hydrogels grafted with different design of oligopeptides under xeno-free culture conditions. (**A**) Morphology of human ES (H9) cells cultivated on PVA hydrogels grafted with various oligopeptides (PVA-BSP-1000, PVA-VN1-1000, PVA-VN1G-1000, PVA-VN2C-1000, PVA-BOP-100, PVA-HBP1-1000, and PVA-HBP2C-1000) and on Synthemax II-coated dishes at passage 1. Scale bar indicates 100 μm. (**B**) Average doubling time of human ES (H9) cells on PVA hydrogels grafted with various oligopeptides (PVA-BSP-500, PVA-BSP-1000, PVA-VN1-500, PVA-VN1-1000, PVA-VN1G-500, PVA-VN1G-1000, PVA-VN2C-500, PVA-VN2C-1000, PVA-BOP-10, PVA-BOP-50, and PVA-BOP-100), Synthemax II-coated dishes, and rVitronectin-coated dishes during passages 1–3. *p < 0.05; **p < 0.05; ***p < 0.05. (**C**) Differentiation ratio of human ES (H9) cells on PVA hydrogels grafted with various oligopeptides (PVA-BSP-500, PVA-BSP-1000, PVA-VN1-500, PVA-VN1-1000, PVA-VN1G-500, PVA-VN1G-1000, PVA-VN2C-500, PVA-VN2C-1000, PVA-BOP-10, PVA-BOP-50, and PVA-BOP-100), Synthemax II-coated dishes, and rVitronectin-coated dishes during passages 1–3.

**Figure 4:**
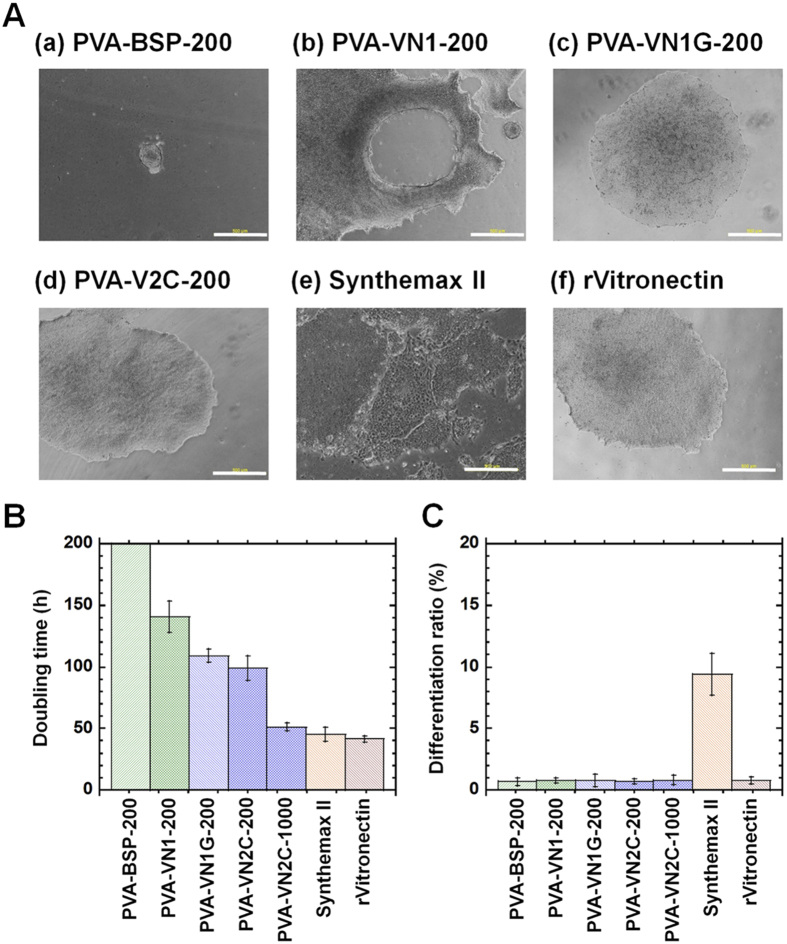
Human ES cells cultured on PVA hydrogels grafted with different design of oligopeptides under xeno-free culture conditions, prepared with a low oligopeptide concentration (200 μg/mL). (**A**) Morphology of human ES (H9) cells cultivated on PVA hydrogels grafted with various oligopeptides (a) PVA-BSP-200, (b) PVA-VN1-200, (c) PVA-VN1G-200, and (d) PVA-VN2C-200), on (e) Synthemax II-coated dishes, and on (f) rVitronectin-coated dishes at passage 1. Scale bar indicates 500 μm. (**B**) Average doubling time of human ES (H9) cells grown on PVA hydrogels grafted with various oligopeptides (PVA-BSP-200, PVA-VN1-200, PVA-VN1G-200, PVA-VN2C-200, and PVA-VN2C-1000), on Synthemax II-coated dishes, and on rVitronectin-coated dishes during passages 1–3. (**C**) Differentiation ratio of human ES (H9) cells grown on PVA hydrogels grafted with various oligopeptides (PVA-BSP-200, PVA-VN1-200, PVA-VN1G-200, PVA-VN2C-200, and PVA-VN2C-1000), on Synthemax II-coated dishes, and on rVitronectin-coated dishes during passages 1–3.

Human ES cells were cultivated on PVA hydrogels grafted with oligopeptides in several different designs. The oligopeptides used in this study were selected by considering different binding mechanisms (i.e., integrin-binding domains [VN1, VN1G, VN2C, BSP, and BOP] and glycosaminoglycan (GAG) binding domains [HBP1 and HBP2C]). Human ES cells (H9) were cultivated on PVA hydrogels grafted with various oligopeptide designs with an elasticity of 25 kPa for passages 1–3. Cell morphology, average doubling time per passage, and average differentiation ratio of human ES cells were investigated during culture for passages 1–3, and the results are shown in Figs. 3 and 4. PVA hydrogels grafted with oligopeptides containing integrin-binding sites (PVA-BSP, PVA-VN1, OVA-VN1G, PVA-VN2C, and PVA-BOP) could support the adhesion and proliferation of human ES cells (Fig. 3A,B). However, the PVA hydrogels containing oligopeptides with GAG binding domains (PVA-HBP1 and PVA-HBP2C) could not sustain the attachment and expansion of human ES cells (Fig. 3A(f and g)). In general, GAG binding surfaces used in cooperation with cyclic-RGD enhanced the attachment of human ES cells^21^. Based on our results, cell culture surfaces containing GAG binding sites without cyclic-RGD did not promote the attachment of human ES cells and iPS cells.

The average doubling time was calculated for human ES cells cultured on oligopeptide-grafted PVA hydrogels (Fig. 3B). The average doubling time of human ES cells cultured on PVA hydrogels grafted with single oligopeptides containing integrin-binding domains (PVA-BSP, PVA-VN1, and PVA-VN1G) prepared from 1000 μg/mL oligopeptide solutions was slightly higher than the doubling time on the corresponding PVA hydrogels prepared from 500 μg/mL oligopeptide solutions (*p* < 0.05). This can be explained by the greater surface density of PVA hydrogels prepared from 1000 μg/mL oligopeptide solutions compared to PVA hydrogels prepared from 500 μg/mL oligopeptide solutions as seen in Fig. 2C. Human ES cells cultured on PVA-VN2C hydrogels with dual chain morphology showed the lowest average doubling time (fastest expansion rate) among the oligopeptide-grafted PVA hydrogels examined in this study. Human ES cells cultured on rVitronectin-coated dishes and Synthemax II-coated dishes exhibited a slightly decreased average doubling time per passage than human ES cells cultured on PVA-VN2C hydrogels. However, this difference was not statistically significant.

Human ES cells cultured on PVA-BOP hydrogels, which have a branched oligopeptide morphology, showed a higher average doubling time than cells grown on PVA-VN2C hydrogels (*p* < 0.05). Unexpectedly, the branched type structure PVA hydrogels (PVA-BOP) did not facilitate the expansion of human ES cells. This is probably due to the extremely low grafting density of the branched type of oligopeptides on the surface of PVA-BOP hydrogels as seen in Fig. 2C.

The average differentiation ratio of human ES cells during passages 1–3 was evaluated for human ES cells cultured on oligopeptide-grafted PVA hydrogels, Synthemax II-coated dishes, and rVitronectin-coated dishes, and the results are shown in Fig. 3C. PVA hydrogels grafted with oligopeptides utilizing an integrin-binding mechanism (PVA-BSP, PVA-VN1, PVA-VN1G, PVA-VN2C, and PVA-BOP) as well as rVitronectin-coated dishes showed extremely low differentiation ratios, indicating that pluripotency of human ES cells was maintained on these hydrogels. In contrast, dishes prepared from the commercially available coating material Synthemax II, which was expected to have the same oligopeptide sequence of VN1 in this study^25^, showed an extremely high differentiation ratio (9%) (Fig. 3C). Human ES cells easily differentiated when cultured on Synthemax II-coated dishes as reported previously^12^, although the average doubling time of human ES cells cultured on Synthemax II-coated dishes was similar or relatively higher than the doubling time of cells grown on oligopeptide-derived PVA hydrogels in this study. This reinforced the importance of considering both physical cues (i.e., elasticity of cell culture materials) and biochemical cues (i.e., oligopeptides of cell binding domain) when maintaining the pluripotency of human ES cells. The Synthemax II-coated dishes were prepared on a stiff TCPS surface (3700 kPa)^2^,^12^, whereas the oligopeptide-grafted PVA hydrogels used in this study were made of relatively soft hydrogels (25 kPa). This suggests that optimal elasticity of cell culture substrates is important for maintaining the pluripotency of human ES cells.

Although PVA hydrogels grafted with oligopeptides derived from vitronectin (PVA-VN1, PVA-VN1G, PVA-VN2C, and PVA-BOP) and bone sialoprotein (PVA-BSP) supported the adhesion and proliferation of human ES cells when prepared with high concentration of oligopeptides (500 and 1000 μg/mL), human ES cells were also cultivated on PVA hydrogels grafted with these oligopeptides (PVA-BSP, PVA-VN1, PVA-VN1G, and PVA-VN2C) prepared with a lower concentration of oligopeptides (200 μg/mL) to evaluate the differences in using PVA hydrogels grafted with these oligopeptides (Fig. 4). Human ES cells hardly attached to PVA hydrogels presenting BSP on the surface (i.e., PVA-BSP-200), whereas human ES cells extensively attached to PVA hydrogels grafted with PVA-VN1-200, PVA-VN1G-200, and PVA-VN2C-200 prepared using 200 μg/mL oligopeptide solutions (Fig. 4A,B). Furthermore, human ES cells easily detached from PVA-VN1-200 hydrogels, indicating a relatively weaker adhesion to this substrate compared to adhesion to PVA-VN1G-200 and PVA-VN2C-200 hydrogels (Fig. 4A), although the grafting densities were similar among these substrates (Fig. 2). On the other hand, human ES cells extensively attached to PVA-VN1G-200 and PVA-VN2C-200 hydrogels prepared with 200 μg/mL oligopeptide solutions, although the grafting concentration of the oligopeptides on the hydrogels was relatively less than the densities of the corresponding PVA hydrogels prepared with 1000 μg/mL oligopeptide solutions (Fig. 2). Figure 4B,C show the average doubling times and average differentiation ratios of human ES cells cultured on oligopeptide-grafted PVA hydrogels prepared with 200 μg/mL of oligopeptide solution, rVitronectin-coated dishes, and Synthemax-coated dishes among passages 1–3. Although human ES cells grown on PVA hydrogels grafted with BSP, VN1, VN1G, and VN2C did not grow as fast as human ES cells cultured on rVitronectin-coated or Synthemax-coated dishes (Fig. 4B), human ES cells cultured on the oligopeptide-grafted PVA hydrogels maintained their pluripotency (low differentiation ratio, Fig. 4C), whereas a higher differentiation ratio of human ES cells was found on Synthemax II-coated dishes compared to that on the oligopeptide-grafted PVA hydrogels prepared with a low concentration (200 μg/mL) of oligopeptide solution (*p* < 0.05). It should be mentioned that human ES cells cultured on PVA hydrogels grafted with VN1G and VN2C showed a decreased doubling time (faster expansion rate) compared to cells grown on PVA hydrogels grafted with VN1 (Fig. 4B). These results demonstrate that the joint segment and dual chain patterns of VN1G and VN2C grafted on PVA hydrogels extensively support the attachment, expansion, and pluripotency of human ES cells.

### Long-term culture of human ES and iPS cells under xeno-free culture conditions

PVA-VN1, PVA-VN1G, and PVA-VN2C hydrogels prepared with 1000 μg/mL of oligopeptide solution were found to be preferable cell culture materials for promoting the proliferation of human ES and iPS cells by selecting cell-adhesion molecules of specific oligopeptides (biological cues) and tuning the elasticity (physical cues, 25 kPa) in the previous sections. Therefore, we investigated the effects of long-term culture (10 passages) of human ES and iPS cells on these optimized PVA hydrogels (PVA-VN1-1000, PVA-VN1G-1000, and PVA-VN2C-1000 hydrogels) in a xeno-free cell culture medium (Essential 8) and the results were compared to human ES and iPS cell growth on commercially available Synthemax II-coated dishes and rVitronectin-coated dishes as control experiments.

**Figure 5:**
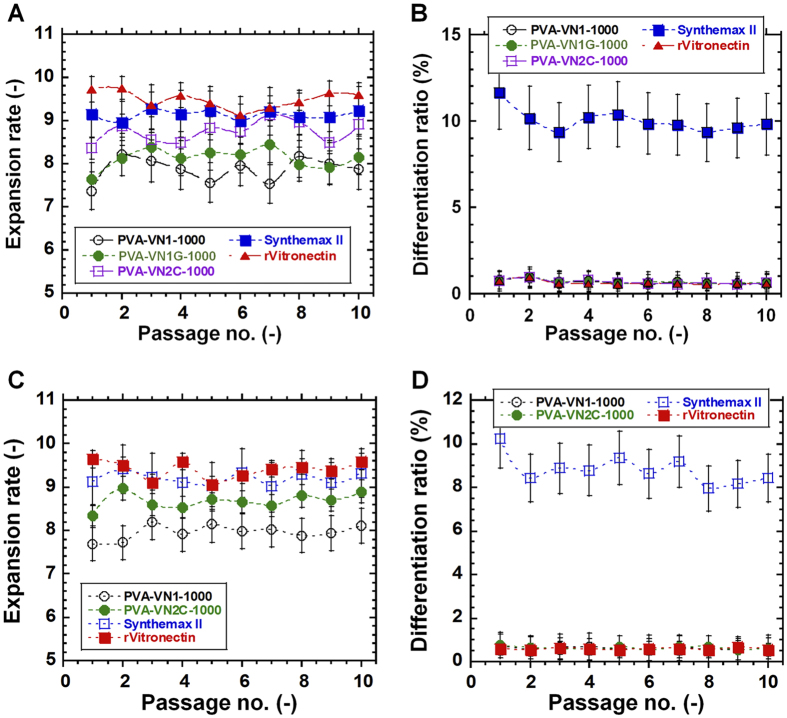
Long-term culture of human ES and iPS cells on PVA hydrogels grafted with different oligopeptide designs under xeno-free culture conditions. (**A**) Expansion rate and (**B**) differentiation ratio of human ES (H9) cells on PVA-VN1-1000 hydrogels (open black circle), PVA-VN1G-1000 hydrogels (closed green circle), PVA-VN2C-1000 hydrogels (open purple square), Synthemax II-coated dishes (closed blue square), and rVitronectin-coated dishes (closed red triangle) for 10 passages. (**C**) Expansion rate and (**D**) differentiation ratio of human iPS (HPS0077) cells on PVA-VN1-1000 hydrogels (open black circle), PVA-VN2C-1000 hydrogels (closed green circle), Synthemax II-coated dishes (open blue square), and rVitronectin-coated dishes (closed red square) for 10 passages.

Figure 5 shows the expansion rates and differentiation ratios of human ES cells (H9) and human iPS cells (HPS0077) cultivated on PVA-VN1-1000, PVA-VN1G-1000, and PVA-VN2C-1000 hydrogels for 10 passages, compared to the cells cultivated on Synthemax II-coated dishes and rVitronectin-coated dishes. The expansion rate of human ES cells on PVA-VN1-1000 and PVA-VN1G-1000 hydrogels was similar (*p* > 0.05), but was slightly less than the expansion rate on PVA-VN2C-1000 hydrogels (*p* < 0.05) (Fig. 5A). Furthermore, these expansion rates of human ES cells on PVA-VN1-1000 and PVA-VNG2-1000 hydrogels were slightly less than expansion rates on Synthemax II-coated dishes and rVitronectin-coated dishes (*p* < 0.05), whereas the expansion rate of human ES cells on PVA-VN2C-1000 hydrogels was not statistically different from the rate of cells grown on Synthemax II-coated dishes (*p* > 0.05).

The expansion rates of human iPS cells cultured on PVA-VN1-1000 and PVA-VN2C-1000 hydrogels showed similar results as the experiments with human ES cells (Fig. 5C). The expansion rate of human iPS cells on PVA-VN2C-1000 hydrogels were not significantly different from the rate of growth on Synthemax II-coated dishes (*p* > 0.05), but was slightly less than the rate of growth on rVitronectin-coated dishes.

Synthemax II-coated dishes and rVitronectin-coated dishes were prepared by coating polymers and proteins on stiff tissue culture polystyrene (TCPS) dishes having an elasticity of 3.7 GPa, whereas PVA-VN1-1000, PVA-VN1G-1000, and PVA-VN2C-1000 hydrogels, which have an elasticity of 25 kPa, are much softer than TCPS dishes. It is generally known that stem cells grow slower on softer materials than on stiffer surfaces^32^. Therefore, the relatively slow growth of human ES and iPS cells observed on the oligopeptide-grafted PVA hydrogels can be attributed to the softness of the hydrogel material compared to the coated TCPS dishes.

Differentiation ratios of human ES cells and iPS cells cultivated on PVA-VN1-1000, PVA-VN1G-1000, and PVA-VN2C-1000 hydrogels as well as rVitronectin-coated dishes were less than 1% as determined by ALP activity, whereas differentiation ratios of human ES cells and iPS cells cultivated on Synthemax II-coated dishes were 8–12% during 10 passages (Fig. 5B,D). Therefore, Synthemax II-coated dishes were not ideal culture materials for maintaining the pluripotency of human ES and iPS cells, whereas PVA-VN1-1000, PVA-VN1G-1000, and PVA-VN2C-1000 hydrogels, and rVitronectin-coated dishes were suitable cell culture materials for maintaining the pluripotency of human ES and iPS cells over time in culture. These results indicate that human ES and iPS cells can be cultured on PVA-VN1-1000, PVA-VN1G-1000, and PVA-VN2C-1000 hydrogels using completely synthetic cell culture materials under feeder-free and xeno-free cell culture conditions, although cells cultured on rVitronectin-coated dishes exhibited relatively faster proliferation (lower doubling time). However, it should be mentioned that rVitronectin is not a synthetic material, rather it is produced by fermentation and extraction from recombinant *E. coli.*, and its production is relatively expensive compared to the cost of producing synthetic materials such as the oligopeptides used to prepare the hydrogels in this study.

**Figure 6:**
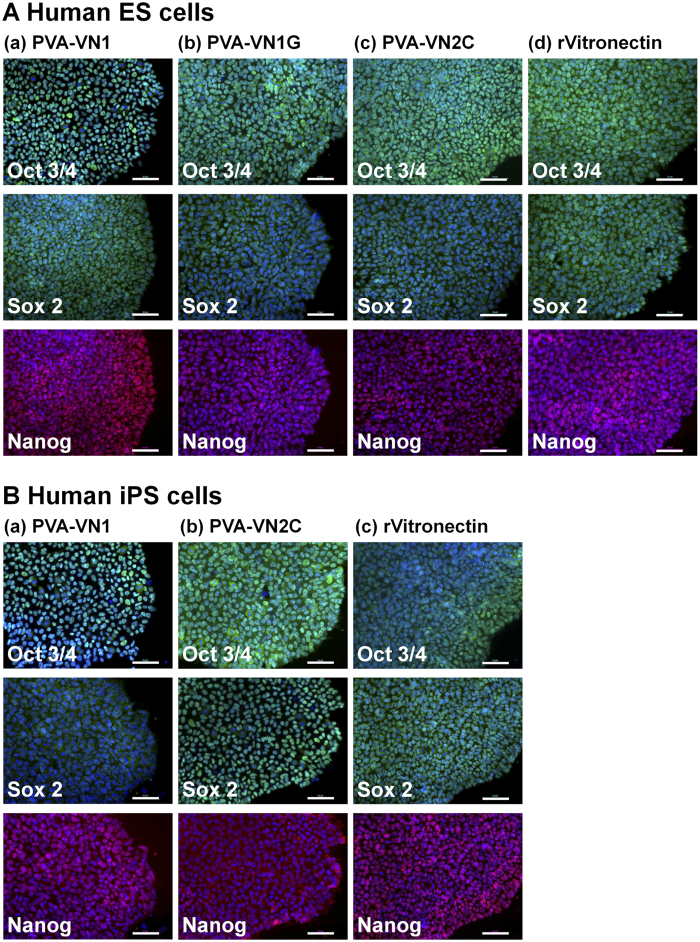
Characterization of the pluripotency of human ES and iPS cells when cultured on PVA hydrogels grafted with different oligopeptide designs under xeno-free culture conditions for 10 passages. (**A**) Expression of pluripotency proteins Oct3/4 (green), Sox2 (green), and Nanog (red) on human ES (H9) cells evaluated by immunostaining with dual staining with Hoechest33342 for nuclear (blue) after culturing on (a) PVA-VN1-1000, (b) PVA-VN1G-1000, and (c) PVA-VN2C-1000 hydrogels, and on (d) rVitronectin-coated dishes under xeno-free conditions for 10 passages. (**B**) Expression of pluripotency proteins Oct3/4 (green), Sox2 (green), and Nanog (red) on human iPS (HPS0077) cells evaluated by immunostaining with dual staining with Hoechest33342 for nuclear (blue) after culturing on (a) PVA-VN1-1000 and (b) PVA-VN2C-1000 hydrogels and on (c) rVitronectin-coated dishes under xeno-free conditions for 10 passages. Scale bar indicates 50 μm.

The pluripotency of human ES and iPS cells was evaluated based on the expression of pluripotent marker proteins (Oct3/4, Sox2, and Nanog) on these cells by immunostaining after culturing on PVA-VN1-1000, PVA-VN1G-1000, and PVA-VN2C-1000 hydrogels as well as on rVitronectin-coated dishes for 10 passages; the results are shown in Fig. 6. These pluripotency proteins were extensively expressed on human ES and iPS cells cultivated on the oligopeptide-grafted hydrogels as well as on rVitronectin-coated dishes in xeno-free conditions for 10 passages.

### Differentiation ability of human ES and iPS cells *in vitro* and *in vivo*

Human ES and iPS cells could be cultivated on PVA hydrogels grafted with several patterns of specific oligopeptides (PVA-VN1-1000, PVA-VN1G-1000, and PVA-VN2C-1000) long term (>10 passages) while maintaining their pluripotency as discussed above. It is also important to evaluate whether these human ES and iPS cells maintained their capacity to differentiate into cells of all three germ layers *in vivo* (teratoma formation assay) and *in vitro* (embryoid body [EB] formation assay) to verify their pluripotency. Human ES cells and human iPS cells were cultivated on PVA-VN1-1000, PVA-VN1G-1000, and PVA-VN2C-1000 hydrogels as well as rVitronectin-coated dishes in xeno-free cell culture conditions for 10 passages, followed by subsequent cultivation in suspension to form EBs.

**Figure 7:**
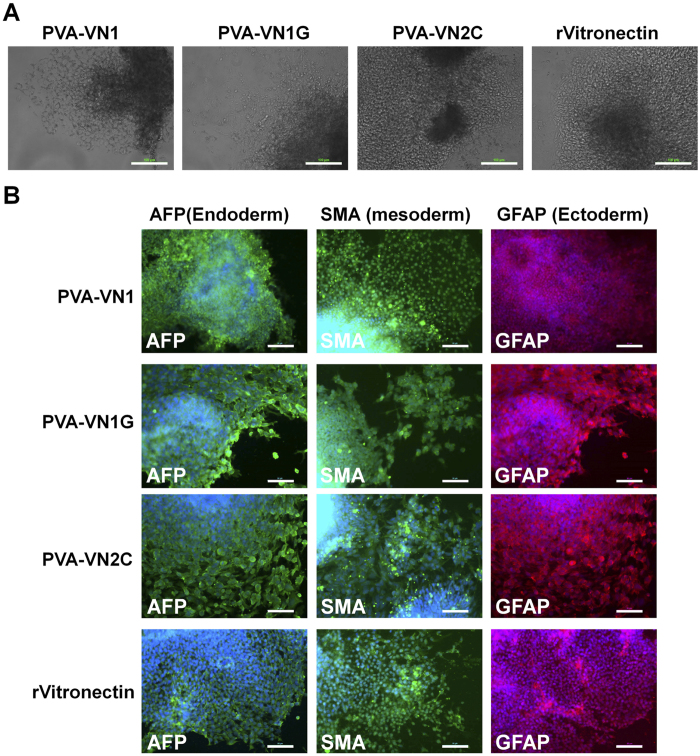
Characterization of the differentiation ability of human ES cells when cultured on PVA hydrogels grafted with different oligopeptide designs under xeno-free culture conditions for 10 passages. (**A**) Morphology of cells from EBs differentiated from human ES (H9) cells after culturing on (a) PVA-VN1-1000, (b) PVA-VN1G-1000, (c) PVA-VN2C-1000 hydrogels, and (d) rVitronectin-coated dishes under xeno-free conditions for 10 passages. Scale bar indicates 100 μm. (**B**) Expression of an endoderm protein (AFP, green), mesoderm protein (SMA, green), and ectoderm protein (GFAP, red) in human ES (H9) cells evaluated by immunostaining with dual staining with Hoechest33342 for nuclear (blue) after culturing on PVA-VN1-1000, PVA-VN1G-1000, and PVA-VN2C-1000 hydrogels, and on (d) rVitronectin-coated dishes under xeno-free conditions for 10 passages. Scale bar indicates 50 μm.

**Figure 8:**
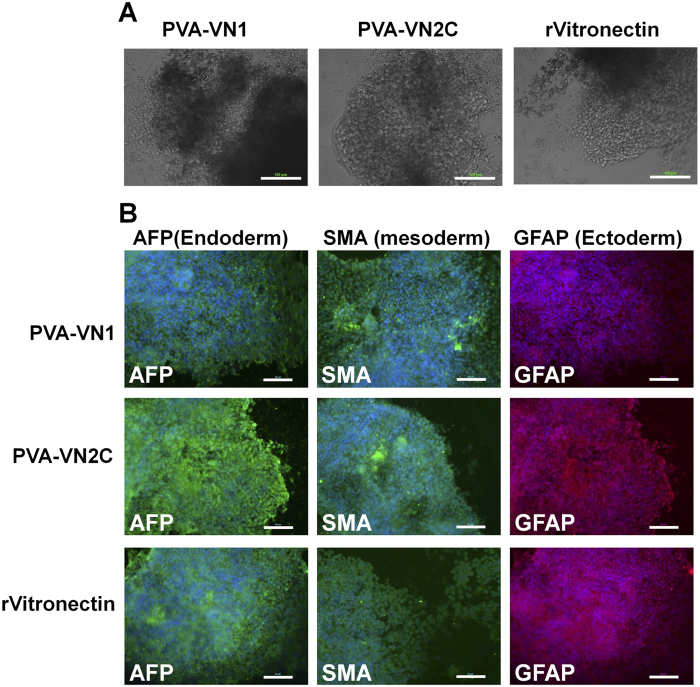
Characterization of the differentiation ability of human iPS cells when cultured on PVA hydrogels grafted with different oligopeptide designs under xeno-free culture conditions after culturing for 10 passages. (**A**) Morphology of cells from EBs differentiated from human iPS (HPS0077) cells after culturing on PVA-VN1-1000 and PVA-VN2C-1000 hydrogels, and on rVitronectin-coated dishes under xeno-free conditions for 10 passages. Scale bar indicates 100 μm. (**B**) Expression of an endoderm protein (AFP, green), mesoderm protein (SMA, green), and ectoderm protein (GFAP, red) in human iPS (HPS0077) cells evaluated by immunostaining with dual staining with Hoechest33342 for nuclear (blue) after culturing on PVA-VN1-1000 and PVA-VN2C-1000 hydrogels, and on rVitronectin-coated dishes under xeno-free conditions for 10 passages. Scale bar indicates 50 μm.

The EBs were further cultured on Matrigel-coated dishes for another 3–4 weeks to observe the ability of the cells to spread on the dishes (Figs 7A and 8A). Differentiated human ES and iPS cells were immunostained for proteins derived from all three germ layers; GFAP (glial fibrillary acidic protein, ectoderm), SMA (smooth muscle actin, mesoderm), and AFP (alpha-fetoprotein, endoderm), and the results are shown in Fig. 7B for human ES cells and Fig. 8B for human iPS cells. Both human ES cells and human iPS cells could differentiate into cells expressing all the aforementioned markers, indicating that the human ES and iPS cells were able to maintain their ability to differentiate into cells from all three germ layers and to maintain their pluripotency *in vitro* after cultivation on PVA-VN1-1000, PVA-VN1G-1000, and PVA-VN2C-1000 hydrogels under xeno-free cell culture conditions.

**Figure 9:**
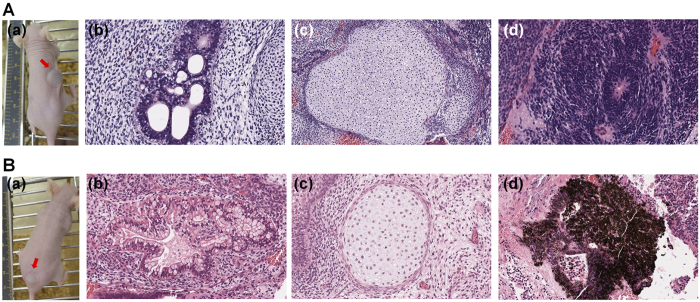
Characterization of the differentiation ability of human ES (H9) cells *in vivo* after culturing on PVA-VN1-1000 and PVA-VN2C hydrogels under xeno-free cell culture conditions for 10 passages. (**A**) (a) A picture of teratoma by injecting with human ES cells after culturing on PVA-VN1-1000 hydrogels after 10 passages. Tissues including (b) intestinal epithelium (endoderm), (c) cartilage (mesoderm), and (d) neuroepithelium (ectoderm) can be observed. (**B**) (a) A picture of teratoma by injecting with human ES cells after culturing on PVA-VN2C-1000 hydrogels after 10 passages. Tissues including (b) intestinal epithelium (endoderm), (c) cartilage (mesoderm), and (d) retinal pigment epithelium (ectoderm) can be observed.

We further investigated the ability of human ES cells to differentiate into cells derived from all three germ layers using an *in vivo* teratoma formation assay. Human ES cells cultivated on PVA-VN1-1000 and PVA-VN2C-1000 hydrogels for 10 passages were subcutaneously xenotransplanted into SCID (non-obese diabetic/severe combined immunodeficiency) mice to yield teratomas *in vivo* (Fig. 9). After the formation, teratomas were isolated from SCID mice and teratoma tissue sections were fixed and stained with H & E (hematoxylin and eosin) and the results are shown in Fig. 9. Teratomas exhibited the presence of cells derived from all three germ layers (neuroepithelium [ectoderm], retinal pigment epithelium [ectoderm], cartilage [mesoderm], and intestinal epithelium [endoderm]). These results indicate that human ES cells cultivated on PVA-VN1-1000, PVA-VN1G-1000, and PVA-VN2C-1000 hydrogels for 10 passages can differentiate into cells of all three germ layers, suggesting that their pluripotency is maintained *in vivo*.

## Discussion

Several cell substrates have been developed for culturing human ES and iPS cells in chemically defined conditions. Generally, ECM-coated dishes and ECM-derived oligopeptide-grafted substrates are used, and ECMs selected for this purpose are usually vitronectin, laminin-332, laminin-521, laminin-511, and fibronectin^15^,^33^,^34^,^35^,^36^,^37^,^38^,^39^,^40^. Natural ECMs are typically isolated from human or animal tissue or blood, whereas recombinant ECMs are typically produced and extracted by fermentation of genetically modified *E. coli*. Due to the expensive manufacturing cost of natural and recombinant ECMs under good manufacturing practices (GMPs), developing substrates of immobilized ECM-derived oligopeptides^11^,^12^,^25^,^41^,^42^,^43^ is important for producing human ES and iPS cells under GMPs for their clinical application.

Recently, synthetic polymers have been reported as acceptable culture substrates for human ES and iPS cells. These heparin-mimicking substrates, PMVE-alt-MA^29^, PMEDSAH^13^,^28^, APMAAm^27^, and copoly(AEtMA-co-DEAEA)^26^, were reported to support human ES and iPS cell culture in chemically defined media. However, a careful investigation is required to find out whether human ES and iPS cells can be cultivated on these synthetic materials, since the attachment mechanism of these stem cells to synthetic polymer substrates is still unclear. Currently, the most reliable synthetic cell culture materials for human ES and iPS cells are immobilized ECM-derived oligopeptides such as PVA-VN2C hydrogels and Synthemax II-coated dishes.

Our results suggest that hydrogel design and surface density of cell-binding domains are important for facilitating the proliferation of human ES and iPS cells and for maintaining the pluripotency of these cells in xeno-free culture conditions for a long-term.

## Materials and Methods

### Materials

Human ES cells (WA09 [H9], WiCell Research Institute, Inc., Madison, WI, USA) and human iPS cells (HPS0077, Riken BioResource Center, Tsukuba, Japan) were used as cell sources. Matrigel (354230, growth factor-reduced basement membrane matrix) was obtained from BD Biosciences (San Jose, CA, USA). Essential 6 medium (A1516401) and Essential 8 medium (A1517001) were obtained from Thermo Fisher Scientific Inc. (Waltham, Massachusetts, USA). TCPS dishes (diameter 35 mm, 35-3001) were purchased from Becton Dickinson (Franklin Lakes, NJ, USA). Poly(vinyl alcohol-co-vinyl acetate-co-itaconic acid) (PVA; AF-17, 1.3 mol% itaconic acid, 98% degree of hydrolysis) was obtained from Japan VAM & Poval Co., Ltd. (Sakai, Osaka, Japan). The oligopeptides of VN1 (KGGPQVTRGDVFTMP), VN1G (GGGGKGGPQVTRGDVFTMP), VN2C (GCGGKGGPQVTRGDVFTMP), BSP (KGGNGEPRGDTYRAY), HBP1 (GKKQRFRHRNRKG), HBP2C (CGGGKKQRFRHRNRKG), and MC (GGGEGGGEGGGEGGG) with purities >98% were obtained from PHJapan (Hiroshima, Japan). Synthemax II-SC (Corning, 3535XX1), N-hydroxysuccinimide (NHS, 13062), and N-(3-dimethylaminopropyl)-N’-ethylcarbodiimidehydrochloride (EDC, 03450) were purchased from Sigma-Aldrich (St. Louis, MO, USA). The alkaline phosphatase detection kit (SCR004) was purchased from EMD Millipore (Billerica, MA, USA). Hoechst 33342 (PA-3014) stain was purchased from Lonza (Basel, Switzerland). Primary antibodies for Oct3/4, Sox2, SSEA-4, TRA-1-81, GFAP (anti-glial fibrillary acidic protein), AFP (anti-alpha-fetoprotein), SMA (anti-smooth muscle actin), and βIII-Tubulin and their secondary antibodies used in this study were purchased from the same companies described in our previous study^12^. All other reagents were obtained from Sigma-Aldrich (St. Louis, MO, USA). Ultrapure water generated by a Milli-Q system (Millipore Corporation, Billerica, MA, USA) was used throughout the experiments.

### Preparation of cross-linked PVA hydrogels grafted with or without oligopeptides

PVA films were prepared from a previously reported method^44^. PVA hydrogels were prepared by crosslinking PVA films with an aqueous crosslinking solution composed of 1.0% (w/v) H_2_SO_4_, 20.0% (w/v) Na_2_SO_4_, and 1.0% (w/v) glutaraldehyde (Fig. 1). The degree of PVA hydrogel crosslinking was controlled by the reaction time. After washing the PVA hydrogels, they were activated with 10.0 mg/mL NHS and 10.0 mg/mL EDC for 6.0 h at 25 °C, followed by immersion of the PVA hydrogels in aqueous solutions containing 200, 500, or 1000 mg/mL oligopeptides to graft the oligopeptides on the PVA hydrogels. The design of oligopeptide-grafted PVA hydrogels is described in Fig. 2. The oligopeptides KGGPQVTRGDVFTMP (VN1), KGGNGEPRGDTYRAY (BSP), and GKKQRFRHRNRKG (HBP1) were grafted on the PVA hydrogels (PVA-VN1, PVA-BSP, and PVA-HBP1, respectively) in single chains. GGGGKGGPQVTRGDVFTMP (VN1G) peptides were grafted on the PVA hydrogels to prepare (PVA-VN1G) hydrogels with single chains and a joint segment (GGGG). Oligopeptides of GCGGKGGPQVTRGDVFTMP (VN2C) derived from a vitronectin cell-binding domain or CGGGKKQRFRHRNRKG (HBP2C) derived from a heparin-binding domain were used to prepare PVA (PVA-VN2C and PVA-HBP2C, respectively) hydrogels grafted with dual chains generated from the spontaneous reaction of S-S (disulfide) bonding upon oxidation by air as these oligopeptides contain cysteine residues. PVA-*X*-*Y* indicates hydrogels grafted with oligopeptide *X* (BSP, VN1, VN1G, VN2C, HBP1, or HBP2C) using *Y* (200, 500, or 1000) μg/mL of the oligopeptide solution.

Branch-type oligopeptide-grafted PVA (PVA-BOP) hydrogels were prepared as follows: a main chain of GGGEGGGEGGGEGGG (MC) containing glutamic acid was grafted on PVA hydrogels using 10, 50, or 100 μg/mL MC solution after the activation with EDC/NHS chemistry as previously described. Subsequently, MC-grafted PVA hydrogels were activated with EDC/NHS chemistry followed by immersion of the hydrogels into 500 μg/mL VN1 oligopeptide solution. PVA-BOP-*Z* indicates hydrogels grafted with the main chain using *Z* (10, 50, or 100) μg/mL solution.

PVA hydrogels grafted with or without oligopeptides were sterilized by immersion in a 75% (v/v) ethanol solution overnight, followed by washing in ultrapure water, and the hydrogels were kept in ultrapure water until use.

### Characterization of PVA hydrogels grafted with or without oligopeptides

The chemical composition (C1s, O1s, and N1s) of the PVA hydrogels grafted with or without oligopeptides was measured using X-ray photoelectron spectroscopy (XPS, K-Alpha spectrometer equipped with a monochromatic Al-K X-ray source, Thermal Scientific, Inc., Amarillo, TX, USA). Data were collected at a photoelectron takeoff angle of 45° with respect to the sample surface. The binding energy (BE) scale was referenced by setting the peak maximum in the C1s spectrum to 284.6 eV.

The storage modulus values of PVA hydrogels prepared from a 5.0% (w/v) PVA solution and crosslinking for 24 h were evaluated using a rheometer (Physica MCR 101, Anton Pars Co. Ltd.) with a 5.0% strain at 1.0 Hz.

### Human pluripotent stem cells (hPSC) culture

The human ES cell line H9 (WA09) and human iPS cell line HPS0077 were maintained on Matrigel-coated dishes in Essential 8 medium using standard protocols. Near-confluent cell clusters were treated with dispase for 1–2 min at 37 °C. The cells were completely dispersed into the medium by repeated pipetting. After centrifugation at 160 × *g* for 5 min at 4 °C, the cells were seeded at the appropriate density (5 × 10^4^ cells per cm^2^) into new culture dishes (e.g., PVA hydrogels grafted with oligopeptides). The medium was changed daily for all the experiments.

### hPSC characterization

The alkaline phosphatase (AP) activity of human ES and iPS cells was evaluated using an alkaline phosphatase detection kit according to the manufacturer’s instructions^12^. Differentiation ratios were calculated for the cells after culturing on the various substrates using the following equation:


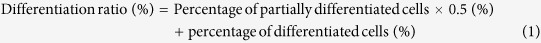


where “differentiated cells” refer to the cells exhibiting no alkaline phosphatase activity and “partially differentiated cells” were defined as the cells exhibiting alkaline phosphatase activity in the center of the colony but not at the edge^12^.

Immunostaining for Nanog, Oct3/4, Sox2, GFAP, AFP, and SMA was performed on human ES and iPS cells to analyze pluripotency and differentiation ability by following a standard protocol^12^. The stained cells were analyzed using fluorescence microscopy (Eclipse Ti-U fluorescence inverted microscope, Nikon Instruments, Inc., Tokyo, Japan).

### Embryoid body formation

The ability of human ES and iPS cells to differentiate into cells derived from three germ layers was investigated by determining embryoid body (EB) formation at passage 10. Human ES and iPS cells were dissociated from the hydrogels, and the cells in the supernatant were collected. Subsequently, the cells were seeded onto ultra-low attachment plates in Essential 6 medium to form EBs. After 14–15 days in suspension culture, EBs were shifted to Matrigel-coated plates and cultivated for an additional 20–30 days. Then, the cells were immunostained with antibodies against differentiation markers (SMA, GFAP, and AFP) of all the three germ layers by following the standard protocol^12^, and the staining was observed using fluorescence microscopy.

### Teratoma formation

The experiments in this study were approved by the ethics committees of the Taiwan Landseed Hospital (IRB-13-05) and the National Central University. All the experiments were performed in accordance with all applicable and relevant governmental and institutional guidelines and regulations for this investigation. Human ES and iPS cells were collected by treatment with a non-enzymatic cell-dissociation solution. After centrifugation, the cell pellets were suspended in DMEM/F12 medium with Matrigel. In total, 5 × 10^6^ cells were injected subcutaneously into non-obese diabetic/severe combined immunodeficiency (SCID) mice. After 5–7 weeks, teratomas had formed, which were subsequently dissected and fixed in formaldehyde solution. Paraffin-embedded teratomas were sectioned and stained with H & E (hematoxylin and eosin) using a standard protocol^12^.

### Statistical analysis

All quantitative results were obtained from four replicates. The data are expressed as the mean ± SD. Statistical analyses were performed using unpaired Student’s *t*-tests in Excel (Microsoft Corporation). Probability values (*p*) less than 0.05 were considered statistically significant.

## Additional Information

**How to cite this article:** Chen, Y.-M. *et al*. Xeno-free culture of human pluripotent stem cells on oligopeptide-grafted hydrogels with various molecular designs. *Sci. Rep.* **7**, 45146; doi: 10.1038/srep45146 (2017).

**Publisher's note:** Springer Nature remains neutral with regard to jurisdictional claims in published maps and institutional affiliations.

## Supplementary Material

Supplementary Information

## References

[b1] Higuchi A. . Biomaterials for the feeder-free culture of human embryonic stem cells and induced pluripotent stem cells. Chem. Rev. 111, 3021–3035 (2009). 10.1021/cr1003612 21344932

[b2] Higuchi A. . Design of polymeric materials for culturing human pluripotent stem cells: Progress toward feeder-free and xeno-free culturing. Prog. Polym. Sci. 39, 1348–1374 (2014).

[b3] Takahashi K. . Induction of pluripotent stem cells from adult human fibroblasts by defined factors. Cell. 131, 861–872 (2007). 18035408 10.1016/j.cell.2007.11.019

[b4] Yu J. . Induced pluripotent stem cell lines derived from human somatic cells. Science 318, 1917–1920 (2007). 18029452 10.1126/science.1151526

[b5] Thomson J. A. . Embryonic stem cell lines derived from human blastocysts. Science 282, 1145–1147 (1998). 9804556 10.1126/science.282.5391.1145

[b6] Burridge P. W. . Chemically defined generation of human cardiomyocytes. Nat. Methods 11, 855–8560 (2014). 24930130 10.1038/nmeth.2999 PMC4169698

[b7] Lian X. . Directed cardiomyocyte differentiation from human pluripotent stem cells by modulating Wnt/beta-catenin signaling under fully defined conditions. Nat. Protoc. 8, 162–175 (2013). 23257984 10.1038/nprot.2012.150 PMC3612968

[b8] Kriks S. . Dopamine neurons derived from human ES cells efficiently engraft in animal models of Parkinson’s disease. Nature 480, 547–551 (2011). 22056989 10.1038/nature10648 PMC3245796

[b9] Pagliuca F. W. . Generation of functional human pancreatic beta cells *in vitro*. Cell 159, 428–439 (2014). 25303535 10.1016/j.cell.2014.09.040 PMC4617632

[b10] Rezania A. . Reversal of diabetes with insulin-producing cells derived *in vitro* from human pluripotent stem cells. Nat. Biotechnol. 32, 1121–1233 (2014). 25211370 10.1038/nbt.3033

[b11] Park H. J. . Bio-inspired oligovitronectin-grafted surface for enhanced self-renewal and long-term maintenance of human pluripotent stem cells under feeder-free conditions. Biomaterials 50, 127–139 (2015). 25736503 10.1016/j.biomaterials.2015.01.015

[b12] Higuchi A. . Long-term xeno-free culture of human pluripotent stem cells on hydrogels with optimal elasticity. Sci. Rep. 5, 18136 (2015). 26656754 10.1038/srep18136 PMC4677349

[b13] Qian X. . Enhancement of the propagation of human embryonic stem cells by modifications in the gel architecture of PMEDSAH polymer coatings. Biomaterials 35, 9581–9590 (2014). 25189518 10.1016/j.biomaterials.2014.08.015 PMC4164049

[b14] Lu H. F. . A defined xeno-free and feeder-free culture system for the derivation, expansion and direct differentiation of transgene-free patient-specific induced pluripotent stem cells. Biomaterials 35, 2816–2826 (2014). 24411336 10.1016/j.biomaterials.2013.12.050

[b15] Prowse A. B. . Long term culture of human embryonic stem cells on recombinant vitronectin in ascorbate free media. Biomaterials 31, 8281–8288 (2010). 20674971 10.1016/j.biomaterials.2010.07.037

[b16] Petrou P. . Safety and clinical effects of mesenchymal stem cells secreting neurotrophic factor transplantation in patients with amyotrophic lateral sclerosis: Results of phase 1/2 and 2a clinical trials. JAMA Neurol. 73, 337–344 (2016). 26751635 10.1001/jamaneurol.2015.4321

[b17] Yoon S. H. . Complete spinal cord injury treatment using autologous bone marrow cell transplantation and bone marrow stimulation with granulocyte macrophage-colony stimulating factor: Phase I/II clinical trial. Stem Cells 25, 2066–2073 (2007). 17464087 10.1634/stemcells.2006-0807

[b18] Schwartz S. D. . Human embryonic stem cell-derived retinal pigment epithelium in patients with age-related macular degeneration and Stargardt’s macular dystrophy: follow-up of two open-label phase 1/2 studies. Lancet 385, 509–516 (2015). 25458728 10.1016/S0140-6736(14)61376-3

[b19] Deng Y. . Long-term self-renewal of human pluripotent stem cells on peptide-decorated poly(OEGMA-co-HEMA) brushes under fully defined conditions. Acta Biomater. 9, 8840–8850 (2013). 23891809 10.1016/j.actbio.2013.07.017

[b20] Higuchi A. . Preparation of induced pluripotent stem cells on dishes grafted on oligopeptide under feeder-free conditions. J. Taiwan Inst. Chem. Eng. 45, 295–301 (2014).

[b21] Klim J. R. . A defined glycosaminoglycan-binding substratum for human pluripotent stem cells. Nat. Methods. 7, 989–994 (2010). 21076418 10.1038/nmeth.1532 PMC2994976

[b22] Chen X. . Thermoresponsive worms for expansion and release of human embryonic stem cells. Biomacromolecules 15, 844–855 (2014). 24571238 10.1021/bm401702h

[b23] Wu S. . Spider silk for xeno-free long-term self-renewal and differentiation of human pluripotent stem cells. Biomaterials 35, 8496–8502 (2014). 25043502 10.1016/j.biomaterials.2014.06.039

[b24] Kolhar P. . Synthetic surfaces for human embryonic stem cell culture. J. Biotechnol. 146, 143–146 (2010). 20132848 10.1016/j.jbiotec.2010.01.016

[b25] Melkoumian Z. . Synthetic peptide-acrylate surfaces for long-term self-renewal and cardiomyocyte differentiation of human embryonic stem cells. Nat. Biotechnol. 28, 606–610 (2010). 20512120 10.1038/nbt.1629

[b26] Zhang R. . A thermoresponsive and chemically defined hydrogel for long-term culture of human embryonic stem cells. Nat. Commun. 4, 1335 (2013). 23299885 10.1038/ncomms2341 PMC3562446

[b27] Irwin E. F. . Engineered polymer-media interfaces for the long-term self-renewal of human embryonic stem cells. Biomaterials 32, 6912–6919 (2011). 21774983 10.1016/j.biomaterials.2011.05.058 PMC3148342

[b28] Villa-Diaz L. G. . Synthetic polymer coatings for long-term growth of human embryonic stem cells. Nat. Biotechnol. 28, 581–583 (2010). 20512122 10.1038/nbt.1631 PMC3471651

[b29] Brafman D. A. . Long-term human pluripotent stem cell self-renewal on synthetic polymer surfaces. Biomaterials 31, 9135–9144 (2010). 20817292 10.1016/j.biomaterials.2010.08.007 PMC2949524

[b30] Braam S. R. . Recombinant vitronectin is a functionally defined substrate that supports human embryonic stem cell self-renewal via alphavbeta5 integrin. Stem Cells 26, 2257–2265 (2008). 18599809 10.1634/stemcells.2008-0291

[b31] Hoffman L. M. & Carpenter M. K. Characterization and culture of human embryonic stem cells. Nat. Biotechnol. 23, 699–708 (2005). 15940242 10.1038/nbt1102

[b32] Winer J. P. . Bone marrow-derived human mesenchymal stem cells become quiescent on soft substrates but remain responsive to chemical or mechanical stimuli. Tissue Eng. Part A 15, 147–154 (2009). 18673086 10.1089/ten.tea.2007.0388

[b33] Chen G. . Chemically defined conditions for human iPSC derivation and culture. Nat. Methods 8, 424–429 (2011). 21478862 10.1038/nmeth.1593 PMC3084903

[b34] Rodin S. . Clonal culturing of human embryonic stem cells on laminin-521/E-cadherin matrix in defined and xeno-free environment. Nat. Commun. 5, 3195 (2014). 24463987 10.1038/ncomms4195

[b35] Rodin S. . Long-term self-renewal of human pluripotent stem cells on human recombinant laminin-511. Nat. Biotechnol. 28, 611–615 (2010). 20512123 10.1038/nbt.1620

[b36] Miyazaki T. . Laminin E8 fragments support efficient adhesion and expansion of dissociated human pluripotent stem cells. Nat. Commun. 3, 1236 (2012). 23212365 10.1038/ncomms2231 PMC3535336

[b37] Yap L. Y. . Defining a threshold surface density of vitronectin for the stable expansion of human embryonic stem cells. Tissue Engin. Part C Methods 17, 193–207 (2011). 10.1089/ten.TEC.2010.0328 20726687

[b38] Rodin S. . Monolayer culturing and cloning of human pluripotent stem cells on laminin-521-based matrices under xeno-free and chemically defined conditions. Nat. Protoc. 9, 2354–2368 (2014). 25211513 10.1038/nprot.2014.159

[b39] Laperle A. . Alpha-5 laminin synthesized by human pluripotent stem cells promotes self-renewal. Stem Cell Rep. 5, 195–206 (2015). 10.1016/j.stemcr.2015.06.009 PMC4618661 26235893

[b40] Badenes S. M. . Defined essential 8 medium and vitronectin efficiently support scalable xeno-free expansion of human induced pluripotent stem cells in stirred microcarrier culture systems. PLoS One 11, e0151264 (2016). 26999816 10.1371/journal.pone.0151264 PMC4801338

[b41] Zhou P. . Simple and versatile synthetic polydopamine-based surface supports reprogramming of human somatic cells and long-term self-renewal of human pluripotent stem cells under defined conditions. Biomaterials 87, 1–17 (2016). 26897536 10.1016/j.biomaterials.2016.02.012

[b42] Wu S. . Efficient passage of human pluripotent stem cells on spider silk matrices under xeno-free conditions. Cell. Mol. Life Sci. 73, 1479–1488 (2016). 26427704 10.1007/s00018-015-2053-5 PMC11108266

[b43] Pennington B. O. . Defined culture of human embryonic stem cells and xeno-free derivation of retinal pigmented epithelial cells on a novel, synthetic substrate. Stem Cells Transl. Med. 4, 165–177 (2015). 25593208 10.5966/sctm.2014-0179 PMC4303358

[b44] Kumar S. S. . The combined influence of substrate elasticity and surface-grafted molecules on the *ex vivo* expansion of hematopoietic stem and progenitor cells. Biomaterials 34, 7632–644 (2013). 23876761 10.1016/j.biomaterials.2013.07.002

