# Supplementary material for: Xeno-free culture of human pluripotent stem cells on oligopeptide-grafted hydrogels with various molecular designs
Source: Sci Rep. 2017 Mar 23;7:45146. doi: 10.1038/srep45146 (PMC5362828; doi:10.1038/srep45146)
Supplement: Supplementary Information [file srep45146-s1.pdf]

Supplementary Material for xeno-free culture of human pluripotent stem cells on oligopeptide-grafted hydrogels with various molecular designs

Yeng-Ming Chen[1,§], Li-Hua Chen[1,§], Meng-Pei Li[1,§], Hsing-Fen Li[1], Akon Higuchi[1,2,3,*], S. Suresh Kumar[4], Qing-Dong Ling[5,6], Abdullah A. Alarfaj[3], Murugan A. Munusamy[3], Yung Chang[7], Giovanni Benelli[8], Kadarkarai Murugan[9,10], Akihiro Umezawa[2]

[1]Department of Chemical and Materials Engineering, National Central University, No. 300, Jhongda RD., Jhongli, Taoyuan, 32001 Taiwan

[2]Department of Reproduction, National Research Institute for Child Health and Development, 2-10-1 Okura, Setagaya-ku, Tokyo 157-8535, Japan

[3]Department of Botany and Microbiology, College of Science, King Saud University, Riyadh 11451, Saudi Arabia

[4]Department of Medical Microbiology and Parasitology, Universiti Putra Malaysia, 43400 Serdang, Slangor, Malaysia

[5]Cathay Medical Research Institute, Cathay General Hospital, No. 32, Ln 160, Jian-Cheng Road, Hsi-Chi City, Taipei, 221, Taiwan

[6]Graduate Institute of Systems Biology and Bioinformatics, National Central University, No. 300, Jhongda RD., Jhongli, Taoyuan, 32001 Taiwan

[7]Department of Chemical Engineering, R&D Center for Membrane Technology, Chung Yuan Christian University, 200, Chung-Bei Rd., Chungli, Taoyuan, 320, Taiwan

[8]Department of Agriculture, Food and Environment, University of Pisa, Via del Borghetto 80, 56124 Pisa, Italy

[9]Division of Entomology, Department of Zoology, School of Life Sciences, Bharathiar University, Coimbatore, Tamil Nadu, 641 046, India

[10]Department of Zoology, Thiruvalluvar University, Serkkadu, Vellore 632 115, India

Correspondence should be addressed to A.H. (higuchi@ncu.edu.tw)

[§] These authors contributed equally to this work.

SUPPLEMENTARY FIGURES

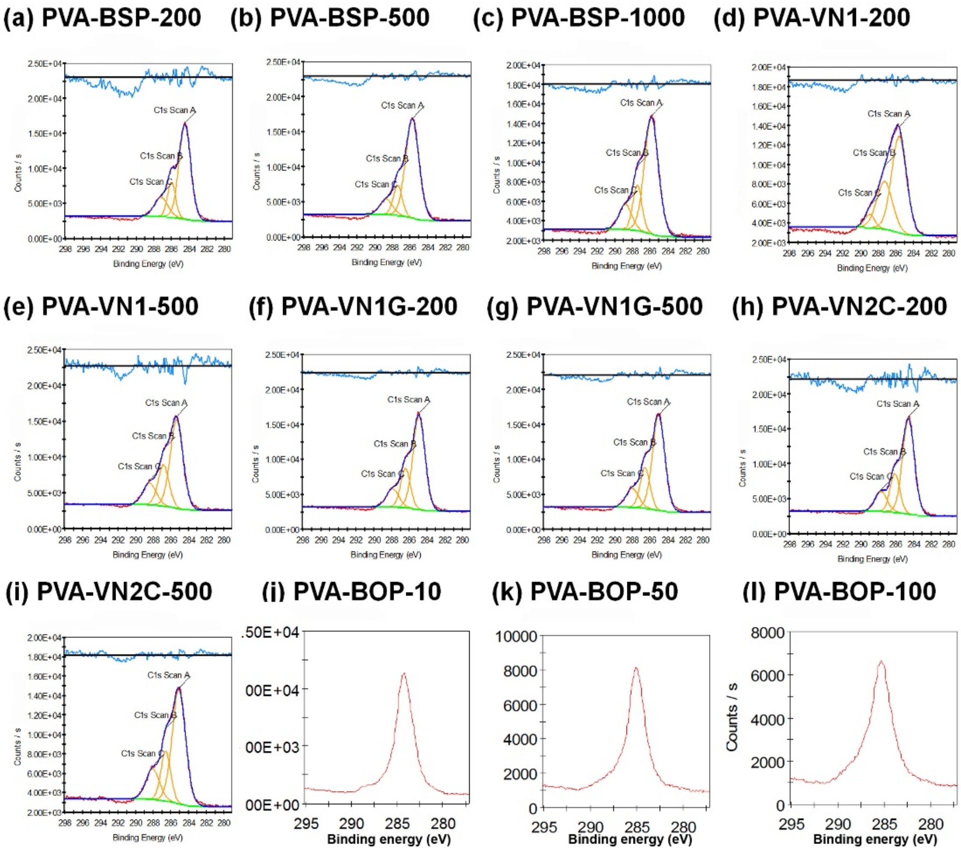

Supplementary Figure 1 Characterization of PVA hydrogels grafted with various oligopeptides. High-resolution XPS spectra of the C1s peaks analyzed on the surface of PVA-BSP-200 (a), PVA-BSP-500 (b), PVA-BSP-1000 (c), PVA-VN1-200 (d), PVA-VN1-500 (e), PVA-VN1G-200 (f), PVA-VN1G-500 (g), PVA-VN2C-200 (h), PVA-VN2C-500 (i), PVA-BOP-10 (j), PVA-BOP-50 (k), and PVA-BOP-100 (l) hydrogels.

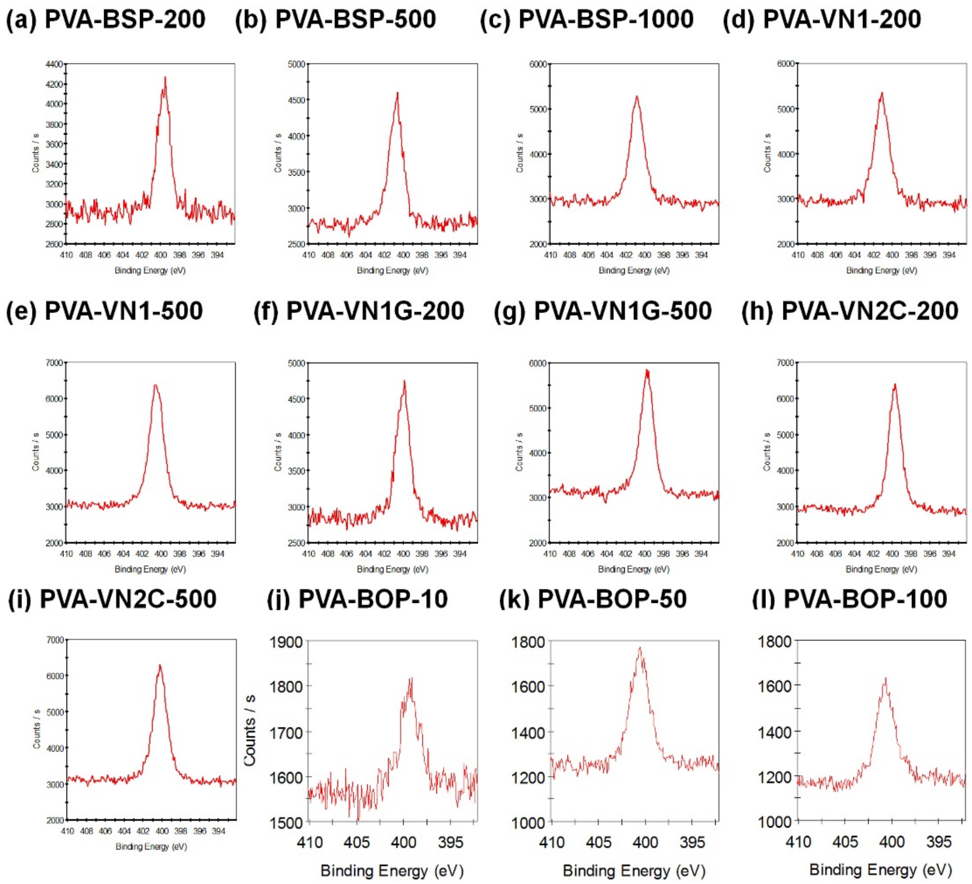

Supplementary Figure 2 Characterization of PVA hydrogels grafted with various oligopeptides. High-resolution XPS spectra of the N1s peaks analyzed on the surface of PVA-BSP-200 (a), PVA-BSP-500 (b), PVA-BSP-1000 (c), PVA-VN1-200 (d), PVA-VN1-500 (e), PVA-VN1G-200 (f), PVA-VN1G-500 (g), PVA-VN2C-200 (h), PVA-VN2C-500 (i), PVA-BOP-10 (j), PVA-BOP-50 (k), and PVA-BOP-100 (l) hydrogels.
